# sEPCR Levels in Chronic Myeloproliferative Diseases and Their Association with Thromboembolic Events: A Case-Control Study

**DOI:** 10.4274/tjh.2012.0150

**Published:** 2014-06-10

**Authors:** Figen Atalay, Selami Koçak Toprak, Ebru Koca, Sema Karakuş

**Affiliations:** 1 Başkent University Faculty of Medicine, Department of Hematology, İstanbul, Turkey; 2 Başkent University Faculty of Medicine, Department of Hematology, Ankara, Turkey

**Keywords:** Myeloproliferative disorders, Endothelial cell protein C receptor, Thrombosis

## Abstract

**Objective: **Venous, arterial, and microcirculatory events are frequently encountered in the clinical course of essential thrombocytosis and polycythemia vera. We aimed to investigate the levels of soluble endothelial protein C receptor (sEPCR) in myeloproliferative diseases to see whether there was a difference between the patients with and without history of thromboembolism.

**Materials and Methods:** The study included patients with polycythemia vera (n=12), patients with essential thrombocytosis (n=13), and controls (n=29). In all groups, we measured proteins C and S, antithrombin and sEPCR levels, and plasma concentrations of thrombin-antithrombin complex, prothrombin fragments 1+2, and D-dimer.

**Results:** Comparing the patients with and without history of thromboembolic attack, statistically significant differences were not observed in terms of sEPCR, D-dimer, thrombin-antithrombin complex, prothrombin fragments 1+2, and hematocrit levels (p=0.318, 0.722, 0.743, 0.324, and 0.065, respectively).

**Conclusion:** Significant increase in the parameters that reflect activation of coagulation, such as sEPCR, thrombin-antithrombin complex, prothrombin fragments 1+2, and D-dimer, reflects the presence of a basal condition that leads to a tendency toward thrombosis development in ET and PV when compared to healthy controls.

## Abstract (tr)

**Amaç:** Esansiyel trombositemi (ET) ve polisitemi vera (PV)’nin klinik seyrinde venöz, arteriyel ve mikrosirkülatuar trombotik olaylara sık olarak rastlanmaktadır. Biz, tromboembolik olay öyküsü olan ve olmayan hastalardaki çözünür endotelyal protein C reseptörü (sEPCR) düzeyleri açısından bir fark olup olmadığını araştırmayı amaçladık.

**Gereç ve Yöntemler:** Çalışmaya polisitemia vera (12) ve esansiyel trombositozu (13) toplam 25 hasta ile kontrol grubunu oluşturmak üzere 29 sağlıklı kişi alındı. Her iki grupta da, protein C, S, antitrombin ve sEPCR ile düzeyleri ile trombin-antitrombin kompleks (TAT), protrombin fragmanı 1+2 ve D-Dimer plazma konsantrasyonları ölçülmüştür.

**Bulgular:**Tromboembolik atak öyküsü olan ve olmayan hastalar karşılaştırıldığında sEPCR, D-dimer, TAT, protrombin fragman 1 +2 (F1+2) ve hematokrit düzeyleri açısından istatistiksel olarak anlamlı fark gözlenmemiştir (sırasıyla p=0,318; 0,722; 0,743; 0,324; 0,065).

**Sonuç:** sEPCR, TAT, F1+2 ve D-Dimer gibi pıhtılaşma aktivasyonunu yansıtan parametrelerinde anlamlı artış görülmesi, parametrelerde literatür ile uyumlu olarark kontrol grubuna göre anlamlı artış gösterilmiş olması ET ve PV hastalarında tromboza eğilim oluşturan bazal bir durumun varlığına işaret etmektedir.

## INTRODUCTION

Protein C is a vitamin K-dependent anticoagulant synthesized largely in the liver that plays an important role in the coagulation system [1]. Activation of protein C is catalyzed by the thrombin-antithrombin (TAT) complex [2]. Activated protein C (aPC), combined with its cofactor, protein S, acts as an anticoagulant, inactivating factor Va and factor VIIIa [3]. In recent years studies have shown that, in addition to its important role in the coagulation system, protein C also has cell protection functions such as maintenance of the vascular barrier (continuity of its integrity), inhibition of apoptosis, and inflammation control [2,4]. The endothelial protein C receptor (EPCR), which is a type of transmembrane glycoprotein found in the endothelium that was first defined by Fukudome and Esmon in 1994, also enables activation of protein C [5]. EPCR augments the activation of protein C by approximately 10 times by binding to protein C on the cell surface [4,6]. The activation of protein C is increased by binding to the EPCR via the TAT complex [7]. EPCR is found on the surface of all vessels, particularly large vessels. Thrombin and inflammatory cytokines induce metalloprotease activity on the cell surface and lead to the production of soluble EPCR (sEPCR). Soluble EPCR is the ligand-binding type of EPCR and can be detected in plasma [4]. Anti-sEPCR has recently been discovered in human plasma [3]. sEPCR inhibits the anticoagulant effect of aPC by inhibiting the attachment of the protein to 

phospholipids and inactivating factor Va. This tends to lead to the development of thrombosis [1].

Essential thrombocytosis (ET) and polycythemia vera (PV) are chronic myeloproliferative diseases (CMPDs). These are clonal hematopoietic stem-cell diseases characterized by an increase in leukocyte, erythrocyte, and platelet production [8]. Venous and arterial thrombosis and microcirculatory events are frequently encountered in the clinical course of ET and PV.

The tendency to develop thrombosis is enhanced in these diseases due to hyperviscosity, qualitative and quantitative abnormalities in blood cells, increased production of thromboxane A2, leukocyte activation, and endothelial damage [9]. Hereditary and acquired thrombophilic states (factor V Leiden mutation analysis, homocysteine levels, presence of antiphospholipid antibodies) have been investigated in patients with CMPDs [10].

In this study, we aimed to investigated the sEPCR levels in CMPDs and to determine potential differences between patients with and without a history of thromboembolism. Only one study to date appears to have investigated the role of levels of natural anticoagulants (protein C, protein S, and antithrombin) in the etiology of thromboembolism in CMPDs; this study detected low levels in patients who had experienced a thrombotic attack [9]. In our study, we to investigated the association of protein C, protein S, antithrombin levels, plasma concentrations of the TAT complex, prothrombin fragments 1+2 (F1+2), D-dimer and sEPCR levels with development of thrombosis in ET and PV patients.

## MATERIALS AND METHODS

**Study Group**

The study included patients with PV (n=12) and ET (n=13) who were over 18 years of age, had been followed in the hematology clinic of Başkent University Medical Faculty for 5 years, and had agreed to participate in the study, as well as 29 healthy volunteers. Nine milliliters of venous blood was collected by venipuncture in tubes containing sodium citrate from all patients and controls. Plasma was obtained by centrifugation at 2300 x g for 10 min at room temperature and immediately stored at -80 °C until use. Soluble EPCR levels were determined in plasma with sEPCR Asserachrom enzyme-linked immunosorbent assay (ELISA) kits (Asserachrom® sEPCR, Diagnostica Stago) according to the manufacturer’s instructions.

Regular C-reactive protein (CRP) levels were studied simultaneously to exclude inflammation. The TAT complex, F1+2, and D-dimer, were analyzed using an ELISA kit (Enzygnost F1+2, Enzygnost TAT, Dade Behring; D-dimer: Asserachrom, Roche Diagnostics). Levels of antithrombin and protein C, which are natural anticoagulants, were quantitatively measured by the colorimetric method (STA, Stachrom Analyzer); total protein S was measured quantitatively by the immunoturbidimetric method (STA, Stachrom Liatest). Informed consent was obtained from all patients and healthy volunteers. The study was started after obtaining the approval of the local ethics committee.

**Statistical**

SPSS 15.0 for Windows was used for statistical analysis. Categorical variables are represented in frequency tables, whereas numerical variables are represented as descriptive statistics [mean, standard deviation (SD), minimum, and maximum]. Cross-table statistics are given for the categorical comparison of the groups, and the chi-square test was used to identify the significance level. Analysis of variance was used for the comparison of more than 2 groups with normally distributed data, and the Kruskal-Wallis test was used for data not distributed normally. The level of statistical significance was considered to be a p-value of less than 0.05.

## RESULTS

**Figure 1:**
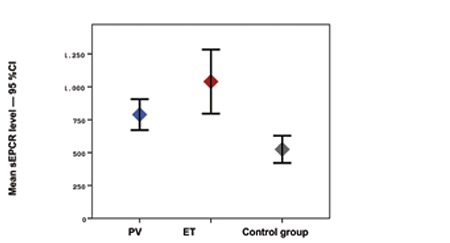
Mean sEPCR levels in PV, ET, and control groups and 95% CI.

**Figure 2:**
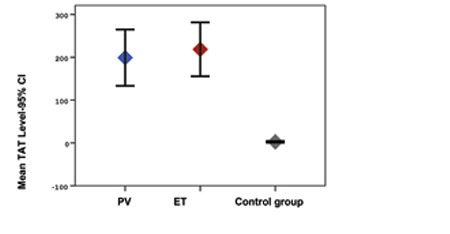
Mean TAT levels in PV, ET, and control groups and 95% CI.

**Figure 3:**
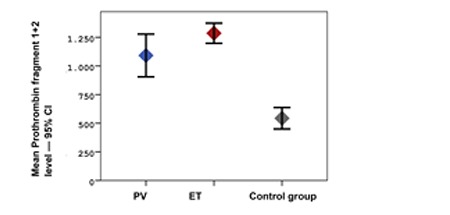
Mean prothrombin fragment 1+2 levels in PV, ET, and control groups and 95% CI.

The study included 25 patients with PV and ET and 29 healthy controls. In the patient group, 48% (n=12) had PV and 52% (n=13) had ET. The mean age ± SD of the patients with PV and ET and of the members of the control group was 64.4±10.4, 68.4±12.1, and 49.5±10.7, respectively (p<0.001). The mean hematocrit level was statistically significantly higher in the control group, whereas the mean platelet, creatinine, CRP, D-dimer, TAT, F1+2, and sEPCR levels were statistically significantly higher in the patient group (Figures 1, 2, and 3). There was no correlation in the statistical comparisons of the protein C, protein S, and antithrombin levels between the patient and the control groups (p=0.821, p=0.984, and p=0.360, respectively). There was a positive correlation between protein C and S levels in patients with ET, whereas there was a negative correlation between protein S and antithrombin levels in the same group (Table 1). There was, however, no association between these levels in patients with PV.

A statistically significant difference was identified between the groups in hematocrit, platelet, creatinine, CRP, D-dimer, TAT, F1+2, and sEPCR levels and in hypertension (HT) and coronary arterial disease (CAD) rates. The mean hematocrit level was higher, whereas CRP and D-dimer levels were lower, in the control group compared to the ET group. The mean platelet, TAT, F1+2, and sEPCR levels were lower in the control group, but the mean creatinine level was higher compared to the ET and PV groups (Table 1). Levels of sEPCR in patients with PV were not correlated with TAT, D-dimer, or F1+2 levels (p=0.656, p=0.137, and p=0.095, respectively). The sEPCR levels in patients with ET were also not correlated with those of TAT, D-dimer, or F1+2 (p=0.946, p=0.852, and p=0.691, respectively) (Table 1). 

In this study, 92% (n=23) of the patients had been receiving antiaggregant therapy and 12% (n=3) had been receiving anticoagulant therapy. Seventy-two percent of the patients had been receiving hydroxyurea. Phlebotomy was performed in 41.7% (n=5) of the patients with PV, whereas the remainder had been receiving either hydroxyurea or interferon therapy. Of the patients with ET, 46.2% (n=6) had been receiving anagrelide and 76.9% (n=10) had been receiving hydroxyurea. Among all patients, the prevalence of cerebrovascular incidents was 12% (n=3) and the prevalence of thromboembolic incidents was 44% (n=11). Arterial thromboses were detected in the majority of the patients (32%, n=8) who developed thrombosis. Most of these were cerebral (24%, n=6) or involved the lower extremities (8%, n=2), abdomen (8%, n=2), or coronary artery (4%, n=1). Four (16%) of the patients who had been receiving antiaggregant therapy developed a second attack, mostly in the form of a cranial thromboembolic event (Table 2). In terms of thromboembolic episodes and the number, location, and type of thromboses, there was no statistically significant difference between patients with ET and PV. Erythromelalgia was found in 2 of the patients with ET, and signs of hyperviscosity were observed in 5 of the patients with ET. Hypermetabolic symptoms were present in 38.5% of the patients. There was no statistically significant difference between the patients with ET and PV in terms of the history of and the number of thromboembolic attacks, or in the type and localization of thrombosis (Table 2).

Comparing the patients with and without a history of thromboembolic attack, no statistically significant difference was observed in the sEPCR, D-dimer, TAT, F1+2, and hematocrit levels (p=0.318, p=0.722, p=0.743, p=0.324, and p=0.065, respectively). However, the difference in the leukocyte and platelet counts was statistically significant (p=0.010 and p=0.027, respectively) (Table 3).

## DISCUSSION

To the best of our knowledge, this is the first study to evaluate levels of sEPCR in ET and PV patients. The mean sEPCR was 524±272 ng/µL in the control group versus 788.8±184 ng/µL in the ET group and 1039.5±403 ng/µL in the PV group. The sEPCR level was statistically significantly higher in the ET and PV patients compared to the control group (p<0.001). Elevated levels of sEPCR reflect a high possibility of a prothrombotic state. However, there was no statistically significant difference between the patients with and without thrombosis in terms of the sEPCR levels (p=0.318). In this study, levels of coagulation activation factors TAT, D-dimer, and F1+2 were also higher in the ET and PV patients compared to the control group (p<0.001, p=0.027, and p<0.001, respectively). These results confirmed enhanced coagulation activation. Unexpectedly, no correlation was seen between levels of sEPCR and these coagulation markers in the ET and PV patient groups. This situation may be attributed to the small number of patients in the trial.

In general, sEPCR has a procoagulant character. It is known that hereditary defects in the protein C system lead to an increase in the propensity to develop venous thromboembolism [11]. It has been reported that the attachment of A23 bp changes the function of EPCR, leading to sequential protein synthesis, which is not expressed on the epithelial surface. However, the role of this mutation in thrombosis is difficult to detect because of its low allele frequency [7,12]. An elevated level of sEPCR also impairs EPCR-mediated coagulation. Studies with healthy subjects demonstrated that the plasma sEPCR level shows a bimodal distribution with age [4,13]. Orhon et al. conducted a study with healthy children and adults and detected an increase in levels of the sEPCR in 20% of children and 10% of adults. The physiological basis of this bimodal distribution is unclear. Genetic factors such as sex and polymorphisms, as well as environmental factors such as smoking and dietary habits, have been considered responsible [13]. The Paris Thrombosis Study investigated genetic risk factors that facilitate venous thromboembolism and compared 338 patients with thrombosis (deep venous thrombosis and/or pulmonary embolus) and a control group comprising an equal number of subjects. The study found that carrying the A3 haplotype is associated with an increase in the level of sEPCR and an increase in the risk for thrombosis [14]. Another study found that the plasma sEPCR level was no lower than 100 ng/mL in pediatric stroke patients with the A3 haplotype, and that the sEPCR level was higher in a stroke group compared to a control group. Based on these results, the authors concluded that the sEPCR level is higher than normal in those with the A3 haplotype, and that this might be associated with a tendency to develop thrombosis [15]. In an analysis of plasma sEPCR levels in 82 patients with retinal venous occlusion, it was observed that the sEPCR level was statistically significantly higher in those with central venous occlusion compared to a control group. The same study failed to detect a statistically significant difference between the time to thrombosis development and the sEPCR level [16]. In another prospective study published recently, the authors concluded that the sEPCR level was high in CAD patients, but there was no significant difference in sEPCR levels between individuals with or without future cardiovascular event [17]. Essential thrombocytosis and PV are clonal hematopoietic stem cell-originated CMPDs associated with an increase in leukocyte, platelet, and erythrocyte production [18,19]. In these patients, thromboembolic complications are the second most frequent cause of mortality after hematological transformation [20]. In the present study, the prevalence of thromboembolic events was 41.7% in the ET patients and 46.2% in the PV patients. These findings are consistent with the literature. Thromboembolic events were mostly cerebral in the PV patients, and lower extremity thrombosis was more common in the ET patients. Although arterial thrombosis was more prevalent in the PV patients, the prevalence of arterial thrombosis and venous thrombosis was the same in the ET patients. None of the patients had been receiving treatment at the time of their first thromboembolic event because they had no known myeloproliferative diseases. All 4 patients who had had a second thrombosis had been receiving treatment. Only 1 patient developed coronary arterial thrombosis during follow-up while receiving treatment. In the literature, the prevalence of thrombosis at the time of diagnosis was 11%-25% in ET patients and 12%-39% in PV patients [21]. Two prospective studies (ECLAP and MRC-PT1) with large patient series reported an annual risk for cardiovascular events for each patient of 2.5%-5% in PV patients and 1.9%-3% in ET patients [22,23]. Although major arterial events (acute myocardial infarction, ischemic stroke, peripheral arterial obstruction, etc.) are more prominent in PV patients, microcirculatory events (erythromelalgia, transient ischemic attack, visual and auditory defects, recurrent headaches, etc.) are encountered more frequently in ET patients. Venous events such as deep venous thrombosis of the lower extremities and pulmonary embolus are frequent in both patient groups [19]. In the ECLAP study, which included 1630 patients with PV, 39% of the patients had a history of thrombosis, and this was the most important cause of mortality (41%). Although an age of over 60 years and a history of previous thrombosis have been considered as the main factors that increase the risk for thrombosis, multivariable analyses have demonstrated that smoking, hypertension, and congestive heart failure might also be associated with an increased risk of thrombosis. The same study demonstrated no relation between the platelet count and thrombosis [22]. Another study demonstrated that acquired aPC resistance occurred in CMPD patients. Decreased levels of protein S and protein C are the probable cause of the prothrombotic state [9,24]. We detected no difference among the PV and ET patients and control group members in terms of levels of protein C, protein S, and antithrombin (p=0.251, p=0.239, p=0.285, respectively). This situation is attributed to the small number of patients in our study.

Leukocytosis was shown to be associated with thrombosis in patients with PV and ET [25]. The mechanism was considered to be an aggregation of platelets and neutrophils after leukocyte activation. On the contrary, in our study leukocyte and platelet counts were found to be significantly lower in patients with thromboembolic attack than in patients without it. This could be secondary to the use of hydroxyurea. Three metaanalyses were published. In these studies, JAK2V617F positivity is associated with increased thrombotic attacks [26,27,28]. At the time of our study, JAK2V617F mutation analysis was not used routinely in our laboratories. It was thus not applied to our patient group.

In conclusion, the lack of a statistically significant difference in the sEPCR levels of the ET and PV patients with and without thrombosis is attributed to the small number of patients in this trial. The significant increases in sEPCR, TAT, F1+2, and D-dimer levels reflect the activation of coagulation and point to the presence of a basal condition that leads to a tendency to develop thrombosis. Large-scale studies with more patients are needed to determine the predictive importance of high levels of sEPCR in the development of thrombotic events in patients with PV and ET.

## CONFLICT OF INTEREST STATEMENT

The authors of this paper have no conflicts of interest, including specific financial interests, relationships, and/or affiliations relevant to the subject matter or materials included.

## Figures and Tables

**Table 1:**
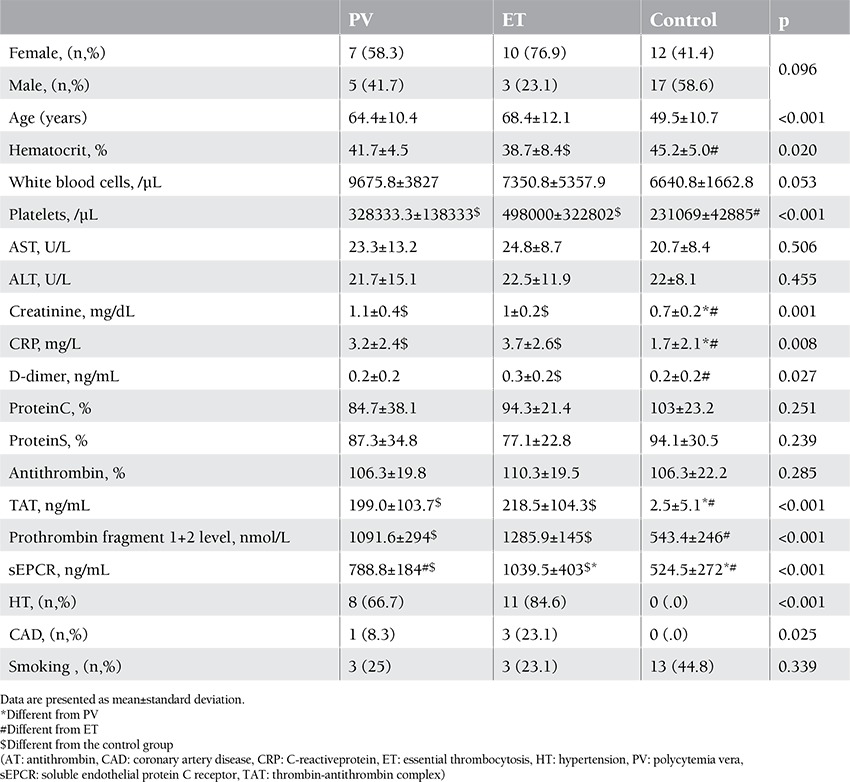
Laboratory parameters in disease control groups.

**Table 2:**
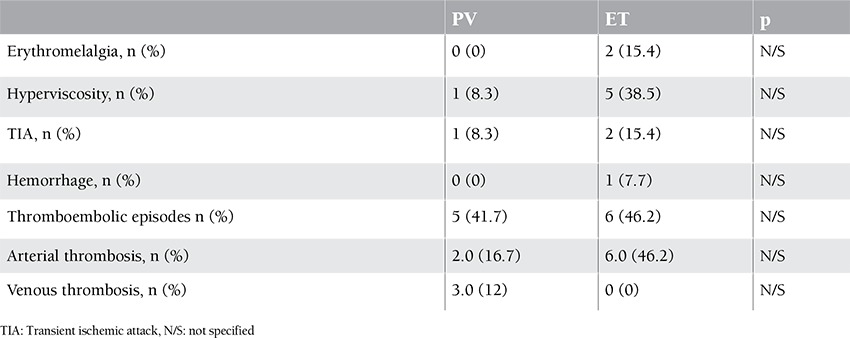
Clinical features of patient groups

**Table 3:**
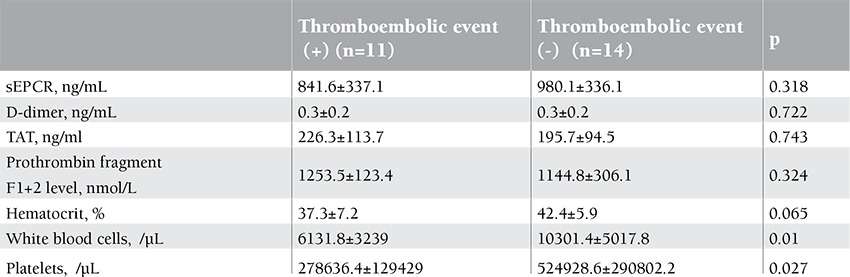
Laboratory values of history with and without thromboembolic event in patients.
